# COVID-19 versus Other Disease Etiologies as the Cause of ARDS in Patients Necessitating Venovenous Extracorporeal Membrane Oxygenation—A Comparison of Patients’ Data during the Three Years of the COVID-19 Pandemic

**DOI:** 10.3390/jcm12216752

**Published:** 2023-10-25

**Authors:** Sua Kim, Hyeri Seok, Beong Ki Kim, Jinwook Hwang, Dae Won Park, Jae Seung Shin, Je Hyeong Kim

**Affiliations:** 1 Department of Critical Care Medicine, Korea University College of Medicine, Korea University Ansan Hospital, Ansan 15355, Republic of Korea; sua0047@gmail.com; 2 Division of Infectious Disease, Department of Internal Medicine, Korea University College of Medicine, Korea University Ansan Hospital, Ansan 15355, Republic of Korea; hyeri.seok@gmail.com (H.S.); pugae1@hanmail.net (D.W.P.); 3 Division of Pulmonology, Department of Internal Medicine, Korea University College of Medicine, Korea University Ansan Hospital, Ansan 15355, Republic of Korea; negero55@korea.ac.kr; 4 Department of Thoracic and Cardiovascular Surgery, Korea University College of Medicine, Korea University Ansan Hospital, Ansan 15355, Republic of Korea; znuke75@korea.ac.kr (J.H.); jason@korea.ac.kr (J.S.S.)

**Keywords:** COVID-19, extracorporeal membrane oxygenation, acute respiratory distress syndrome, prognosis

## Abstract

Considering the characteristics of coronavirus disease 2019 (COVID-19) acute respiratory distress syndrome (ARDS), we compared the clinical course and outcomes of patients with ARDS who received venovenous extracorporeal membrane oxygenation (VV ECMO) based on the etiology of ARDS. This retrospective single-center study included adult patients with severe ARDS necessitating VV ECMO during the COVID-19 pandemic. Among 45 patients who received VV ECMO, 21 presented with COVID-19. COVID-19 patients exhibited lower sequential organ failure assessment scores (9 [8–12.75] versus 8 [4–11.5], *p* = 0.033) but longer duration of VV ECMO support (10.5 days [3.25–29.25] versus 28 days [10.5–70.5] *p* = 0.018), which was accompanied by an weaning off rate from VV ECMO in 12/24 (50%) versus 12/21 (57.1%) and 28-day mortality in 9/24 [37.5%] versus 2/21 [9.5%] in non-COVID-19 and COVID-19 patients (*p* = 0.767, *p* = 0.040), respectively. Finally, in the adjusted Cox regression model for hospital mortality, the hazard ratio of COVID-19 was not significant (hazard ratio 0.350, 95% confidence interval 0.110–1.115, *p* = 0.076). Although the VV ECMO period was longer, COVID-19 did not significantly impact ECMO weaning off and mortality rates. Nonetheless, judicious patient selections based on risk factors should be followed.

## 1. Introduction

Since the 1918 Spanish influenza pandemic [1,2], several waves of viral respiratory diseases have threatened patients and influenced the healthcare system, leading to the evolution of treatments for the diseases and approaches for managing chaotic situations [3]. The use of venovenous extracorporeal membrane oxygenation (VV ECMO) in adult patients with severe acute respiratory distress syndrome (ARDS) exponentially increased during the 2009 H1N1 influenza A pandemic [4], demonstrating an improved survival rate compared to patients receiving conventional treatment [5]. Subsequently, VV ECMO has been used in patients with severe ARDS. When confronted with the coronavirus disease 2019 (COVID-19), we introduced VV ECMO as a rescue device for treatment. Extracorporeal membrane oxygenation (ECMO) played a crucial role, resulting in better survival compared to patients who receive conventional treatment only [6,7].

However, the characteristics of severe ARDS in patients with COVID-19 exceeded our expectations. Variants of the virus influence response to treatment [8]. Pulmonary fibrosis [9,10] caused prolonged VV ECMO support [11,12]. Consequently, during a pandemic, when resources are strained, patient selection and treatment strategies should be employed for those receiving VV ECMO, which is a resource-consuming intervention [4].

We evaluated patients with ARDS who received VV ECMO support during the three years of the COVID-19 pandemic. We compared disease characteristics and clinical outcomes based on the presence of COVID-19. We then identified and assessed significant factors related to the final outcomes of these patients.

## 2. Materials and Methods

### 2.1. Study Population

This was a retrospective single-center study. We screened adult patients who were admitted to the intensive care unit (ICU) of Korea University Ansan Hospital between March 2020 and February 2023 with a diagnosis of severe ARDS and received VV ECMO for the management of ARDS. Severe ARDS was diagnosed based on the Berlin definition: clinical insult or worsening respiratory symptoms combined with bilateral chest opacities and hypoxemia with a PaO_2_/FiO_2_ ratio (PFR) < 100 mmHg that cannot be explained by cardiac failure or fluid overload [13]. Patients were excluded from the study if they encountered complications that prevented the successful implementation of VV ECMO or if the influence of heart failure was assumed to be the primary reason for the patients’ oxygen requirement. 

### 2.2. Data Collection

Demographic parameters included age, sex, medical history, Charlson Comorbidity Index (CCI), Acute Physiology and Chronic Health Evaluation (APACHE) II score, and Sequential Organ Failure Assessment (SOFA) score. We recorded the patients’ condition before VV ECMO cannulation, including mechanical ventilator setting, oxygen requirement, and results of arterial blood gas analysis, which were acquired from electronic medical records. The PFR was calculated using these data. Additionally, we checked the number of days between ICU admission and mechanical ventilation and the number of days between the diagnosis of COVID-19 and VV ECMO initiation. 

### 2.3. VV ECMO Management

Patients with severe ARDS were considered for VV ECMO when hypoxemia was refractory with a PaO_2_ level less than 60 mmHg or hypoxia deteriorated during optimal medical management by multi-disciplinary team that consisted of intensivists in charge of medical ICU, attending physicians of infectious medicine and cardiothoracic surgeons during the COVID-19 pandemic. There was no absolute contraindication except for active major bleeding, severe acute neurologic injury, or ongoing multiorgan failure. Anticoagulation was started using unfractionated heparin when ECMO cannulation is started. During ECMO, multi-disciplinary team rounding was performed for patient care. Our hospital applied the same protocol for VV ECMO both in COVID-19 and non-COVID-19 patients. 

### 2.4. Outcome of the Study

The primary outcome of this study was hospital mortality. The secondary outcomes were weaning off VVECMO, the duration of VV ECMO and hospitalization, and ECMO complications.

### 2.5. Statistical Analysis

Continuous variables were expressed as mean ± standard deviation or median and interquartile range (IQR). Categorical variables are expressed as frequencies and percentages. Continuous variables were compared between groups using the Wilcoxon rank-sum test. Categorical variables were compared using the χ^2^ test. To assess the cumulative mortality rates and the cumulative VV ECMO weaning off rate in each group, a Kaplan–Meier curve was employed, and the log-rank test was used for comparison. Cox proportional hazard regression analysis was performed to evaluate the impact of COVID-19 on hospital mortality. For multivariate analysis, parameters that showed significance in the univariate analysis and parameters that were clinically relevant and required consideration in this study were incorporated. Cases with hopeless weaning off ECMO were regarded as failed weaning cases. Statistical analyses were performed using SPSS version 20.0 (IBM Corp., Armonk, New York, USA) and MedCalc version 22 (MedCalc Software, Ostend, Belgium).

## 3. Results

### 3.1. Baseline Characteristic and Patients’ Condition before VV ECMO in Non-COVID-19 and COVID-19 ARDS Patients

There were 48 patients who underwent VV ECMO during the study period, and three patients were excluded due to suspicion of hydrostatic edema resulting from heart failure, diagnosis of pulmonary thromboembolism, and death on the day of ECMO cannulation with cannula site bleeding. Therefore, 45 patients were enrolled in this study. Among the enrolled patients, 27 were male (60%), the mean age was 61.0 ± 9.5 years, and 21 (46.7%) were diagnosed with COVID-19. The SOFA score was 9.0 (7.0–12.0), and the APACHE II Score was 18.0 (14.0–22.5). Before VV ECMO initiation, the mean positive end-expiratory pressure (PEEP) was 10 (7.75–10.00) cmH_2_O, tidal volume (VT) was 6.78 (6.15–7.83) mL/kg of predicted body weight, and PFR was 59.50 (48.25–78.90) mmHg. 

When demographic data and patient conditions before ECMO were compared between patients with and without COVID-19, there were no significant differences in age, sex, and CCI. However, immunocompromised hosts, including patients with malignancies, were more common in non-COVID-19 patients (*p* = 0.026). The cause of ECMO in non-COVID-19 patients was mostly bacterial infection (n = 11, 45.8%) or pneumonia due to other organisms (n = 4, 16.7%). In terms of patient severity, the SOFA score was lower in COVID-19 patients (9 [8–12.75] versus 8 [4–11.5], *p* = 0.033). Although it was not statistically significant, the requirement of vasopressors assessed using the vasopressor-inotropic score was greater in non-COVID-19 patients (23.7 [21.9–26.9] vs. 3.0 [0.00–10.00], *p* = 0.551). The PFR before ECMO did not differ significantly between the groups (58.9 [44.5–76.6] versus 61.0 [50.7–87.95], *p* = 0.488), but PaCO_2_ was higher in non-COVID-19 patients than COVID-19 patients (63.2 [43.4–71.3], versus 44.0 [37.3–52.6], *p* = 0.006) without a difference in VT/Kg (*p* = 0.481) (Table 1). 

### 3.2. Disease Course and Prognosis of Non-COVID-19 and COVID-19 Patients

In patients with COVID-19, the days from diagnosis of COVID-19 to ECMO initiation was 11 (5.5–19.5). Most patients were intubated within one day of ICU admission (n = 14, 63.6%), and the number of days from mechanical ventilation to ECMO was 3.0 (1.0–10.5). In non-COVID-19 patients, the duration of mechanical ventilation before ECMO was 1.50 (0.00–6.00) days, which was numerically shorter than that of COVID-19 patients but did not differ significantly (*p* = 0.196). 

**Figure 1:**
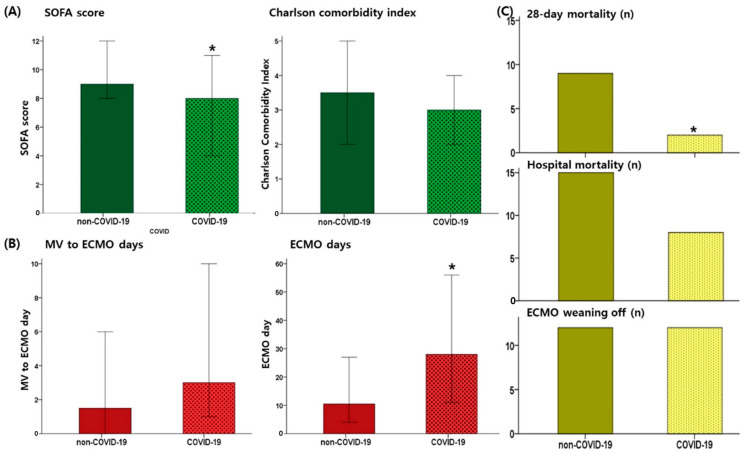
Comparison of patients’ characteristics classified by the diagnosis of COVID-19 as the cause of acute respiratory distress syndrome. (**A**) Sequential organ failure assessment score and Charlson comorbidity index; (**B**) treatment course explained with the time period of the initiation of mechanical ventilation to extracorporeal membrane oxygenation (ECMO) and duration of ECMO support (days); (**C**) outcomes of patients, 28-day mortality, hospital mortality, and the number of patients successfully weaned off ECMO. *, *p* < 0.05 when compared between non-COVID-19 patients and COVID-19 patients. Non-COVID-19 patients were indicated with dotted bar and COVID-19 patients were indicated with solid bar in the graphs.

Finally, 24 of the 45 patients (53.3%) were successfully weaned off VV ECMO, and median duration of VV ECMO was 15 (8.0–45.0) days. The weaning off rate did not differ between non-COVID-19 and COVID-19 patients (12/24 [50.0%] vs. 12/21 [57.1%], *p* = 0.767). However, the duration of ECMO was longer in COVID-19 patients (10.5 days [3.25–29.25] versus 28.0 days [10.5–70.5], *p* = 0.018) (Figure 1, Table 2).

### 3.3. VV ECMO Weaning Off, Mortality, and COVID-19

**Figure 2:**
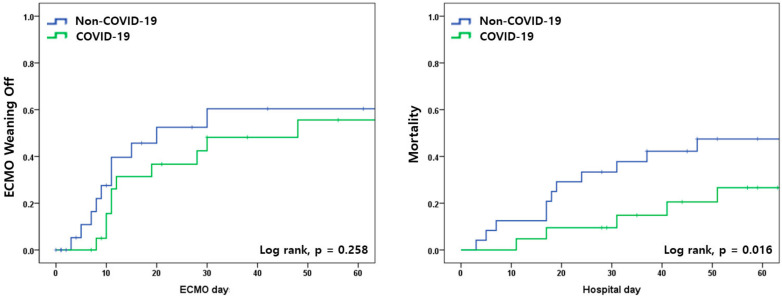
Kaplan–Meier curves for extracorporeal membrane oxygenation (ECMO) weaning off (**left**) and mortality (**right**) rates in non-COVID-19 and COVID-19 patients.

In patients with COVID-19 with a longer duration of ECMO support, the 28-day mortality was lower than that noted in non-COVID-19 patients (9/24 [37.5%] versus 2/21 [9.5%], *p* = 0.040); however, 11 patients among 19 (57.8%) 28-day survivors were supported by ECMO for longer than 28 days. This finding was reflected in Kaplan-Meier curves as a lower ECMO weaning off rate of COVID-19 patients, despite the lack of statistical significance (*p* = 0.258), and lower mortality (*p* = 0.016) simultaneously (Figure 2). 

Finally, hospital mortality was noted in 15 (62.5%) in non-COVID-19 patients and 8 (38.1%) in COVID-19 patients (*p* = 0.139). In the univariate Cox proportional hazard regression analysis for hospital mortality, CCI, SOFA score, APACHE II score, PEEP before VV ECMO, and the diagnosis of COVID-19 were significantly associated with hospital mortality. However, when adjusting for these factors in the multivariate analysis, COVID-19 was not a significant factor for hospital mortality (hazard ratio 0.350, 95% confidence interval 0.110–1.115, *p* = 0.076). Age, CCI, and SOFA score were significantly associated with hospital mortality in multivariate Cox proportional hazard regression analysis (Table 3).

### 3.4. Complications during VV ECMO

During ECMO, one (2.2%) patient experienced intracranial hemorrhage and died of bleeding. Nine patients (20%) experienced gastrointestinal hemorrhage, necessitating endoscopic evaluation and treatment. Two patients (4.4%) developed cannula-related infections, and four patients experienced thrombosis of the ECMO cannula. Finally, 20 patients (44.4%) started continuous renal replacement therapy, and nine patients (20%) experienced pneumothorax during ECMO. Among these, continuous renal replacement therapy was significantly associated with hospital mortality (*p* = 0.007).

## 4. Discussion

We evaluated patients with ARDS who received ECMO support over three years during the COVID-19 pandemic and compared their disease characteristics and clinical outcomes based on the diagnosis of COVID-19 as the cause of ARDS. Patients with COVID-19 exhibited a lower SOFA score and vassopressor-inotropic score on the day of ECMO cannulation but required a longer recovery time for ECMO weaning off than non-COVID-19 patients. Finally, 28-day mortality was lower in COVID-19 patients, but there was no significant difference in the ECMO weaning off rate and hospital mortality between COVID-19 and non-COVID-19 patients in simple analysis. When the effect of COVID-19 on hospital mortality was evaluated by adjusting other influences on ECMO support, COVID-19 was not significant factor. Age, comorbidities, CCI, and SOFA scores on the day of ECMO cannulation were significantly associated with hospital mortality.

### 4.1. Severe ARDS in COVID-19 and VV ECMO

The COVID-19 pandemic has significantly impacted the use of VV ECMO [3]. Numerous reports have been published that provide extensive information on the outcomes of VV ECMO in COVID-19 patients and the risk factors for survival [14]. Although some findings have been inconclusive, most studies agreed that VV ECMO could be beneficial in patients with severe ARDS caused by COVID-19 [6]. Particularly, VV ECMO was found to be advantageous in cases of very severe ARDS with a PFR < 80 mmHg, and, in addition, increased age, the specific period of the COVID-19 pandemic, and the volume of ECMO cases in the center have been identified as important factors affecting mortality [6,15,16,17]. The effect of the durations of VV ECMO support and pre-ECMO mechanical ventilation on mortality seems still controversial [7,15]. In addition, there has been a growing emphasis on the importance of prone positioning in the management of these patients [18,19,20]. As research continues, optimized patient care and improved survival rates are expected with a more comprehensive understanding of the disease.

### 4.2. COVID-19 and Other Etiologies as the Cause of Severe ARDS Requiring ECMO

Recently, several waves of viral infections have caused severe ARDS. The H1N1 pandemic has increased the use of VV ECMO, making it a reasonable treatment option for adults with severe ARDS [5,21]. Thereafter, the use of VV ECMO in adult patients increased, leading to an anticipated improvement in ECMO handling skills. However, the outcomes of COVID-19 patients supported by VV ECMO remain challenging. The mortality rates of these patients were higher than those of patients with H1N1 or other viral pneumonia that caused severe ARDS requiring ECMO support, and the duration of ECMO support in COVID-19 patients was longer [22]. When patients with COVID-19 were compared with patients with various disease etiologies other than viral pneumonia, the duration of ECMO was still longer, but without the difference in survival [2,11,12,18]. These findings highlight the unique challenges posed by COVID-19-related ARDS.

### 4.3. Comparison of ARDS Due to COVID-19 and Other Etiologies in Our Study Patients

In our study, although not statistically significant, patients with COVID-19 had fewer comorbidities. In particular, the number of immunocompromised hosts was significantly lower, and a lower dose of vasopressor was required. These factors were accompanied by the significantly lower SOFA score compared to non-COVID-19 patients. These imply that patients with severe ARDS caused by COVID-19 requiring VV ECMO may be afflicted by prominent lung injury rather than multi-organ impairment due to the systemic inflammatory response. However, this lung injury was followed by a longer time to weaning off VV ECMO. The prolonged VV ECMO support is not unique to these patients as mentioned above [11,23,24], which is consistent with the delayed onset of ARDS or slow progression of the disease in COVID-19. Several studies reported a longer period of ARDS onset from the diagnosis of COVID-19 that exceeded 7 days indicated in Berlin definition for ARDS [13,25,26]. The progression of ARDS was also slower in severe ARDS cases caused by COVID-19 when histopathologic exam was performed. The three phases of ARDS were reported with a longer duration of illness than that documented in H1N1 influenza cases [27,28]. The ECMO duration for patients with COVID-19 in this study may seem considerably longer than previously reported values for COVID-19 patients. Nonetheless, despite receiving VV ECMO support from the same physicians during the identical timeframe, non-COVID-19 patients had a significantly shorter period of VV ECMO support. This suggests that the longer ECMO support in COVID-19 patients in our study may be specific to the disease. It can be supported with the fact that COVID-19 was significant factor related with poor ECMO weaning off in our multivariate Cox proportional hazard regression analysis adjusting for age, sex, SOFA score, and CCI with a hazard ratio of 0.296, 95% confidence interval 0.107–0.817, and *p* = 0.019; which was not reported in main results. Additionally, we hypothesized that the longer ECMO support in COVID-19 patients could have been exaggerated by governmental policies at that period, as medical expenses for COVID-19 care were supported by the government. 

In our study, although not statistically significant, patients with COVID-19 had fewer comorbidities. In particular, the number of immunocompromised hosts was significantly lower, and a lower dose of vasopressor was required. These factors were accompanied by the significantly lower SOFA score compared to non-COVID-19 patients. These imply that patients with severe ARDS caused by COVID-19 requiring VV ECMO may be afflicted by prominent lung injury rather than multi-organ impairment due to the systemic inflammatory response. However, this lung injury was followed by a longer time to weaning off VV ECMO. The prolonged VV ECMO support is not unique to these patients as mentioned above [11,23,24], which is consistent with the delayed onset of ARDS or slow progression of the disease in COVID-19. Several studies reported a longer period of ARDS onset from the diagnosis of COVID-19 that exceeded 7 days indicated in Berlin definition for ARDS [13,25,26]. The progression of ARDS was also slower in severe ARDS cases caused by COVID-19 when histopathologic exam was performed. The three phases of ARDS were reported with a longer duration of illness than that documented in H1N1 influenza cases [27,28]. The ECMO duration for patients with COVID-19 in this study may seem considerably longer than previously reported values for COVID-19 patients. Nonetheless, despite receiving VV ECMO support from the same physicians during the identical timeframe, non-COVID-19 patients had a significantly shorter period of VV ECMO support. This suggests that the longer ECMO support in COVID-19 patients in our study may be specific to the disease. It can be supported with the fact that COVID-19 was significant factor related with poor ECMO weaning off in our multivariate Cox proportional hazard regression analysis adjusting for age, sex, SOFA score, and CCI with a hazard ratio of 0.296, 95% confidence interval 0.107–0.817, and *p* = 0.019; which was not reported in main results. Additionally, we hypothesized that the longer ECMO support in COVID-19 patients could have been exaggerated by governmental policies at that period, as medical expenses for COVID-19 care were supported by the government. 

Meanwhile, the final weaning rate from VV ECMO did not differ between the groups, and patients with COVID-19 and prolonged ECMO ultimately survived. The prognosis of COVID-19 patients in this study was not worse even with the longer period of ECMO. Older age and higher CCI and SOFA scores were associated with increased mortality rates. The development of acute kidney injury requiring continuous renal replacement therapy during ECMO is another factor that affects hospital mortality. This suggests that extended ECMO duration should not be a determinant of treatment in COVID-19 patients; rather, the overall patient condition should be considered when deciding on long-term ECMO support. 

### 4.4. Limitations

This was a retrospective single-center study, and the number of patients included in this study was small. Instead, our patients in both categories underwent VV ECMO during the same period of 3 years during the COVID-19 pandemic. The quality of care and experience of the staff for VV ECMO did not differ between the groups. Therefore, the comparison of patient outcomes between COVID-19 patients and non-COVID-19 patients is more significant in this study. Second, very few patients underwent prone positioning in this study population, which was inevitable during the pandemic period due to the shortage of medical personnel. Third, the non-COVID-19 patient group comprised patients with various etiologies. Although the main cause of ARDS was bacterial pneumonia, and patients with respiratory failure from cardiac origin were excluded, patients with trauma and interstitial lung disease were included in this group. Finally, the indication for ECMO was not as strict as that followed in randomized controlled trials because this study retrospectively evaluated patient outcomes. Although we used the timeline, it was hard to follow strictly due to the rapidly changing nature of the patients’ conditions, especially for COVID-19 patients.

## 5. Conclusions

In this study, we compared patient characteristics, the natural course of the disease, and patient outcomes, including ECMO weaning off and survival, between patients with severe ARDS caused by COVID-19 and other etiologies who received VV ECMO support during the COVID-19 pandemic. Patients with COVID-19 required a longer time to wean off VV ECMO but exhibited a similar weaning off rate. Finally, in the adjusted Cox regression model, COVID-19 did not significantly affect hospital mortality, but age, comorbidities, and severity at baseline did. This implies that prolonged ECMO support for patients with COVID-19 is worth providing; however, a comprehensive evaluation of individual patient conditions is essential for well-informed treatment decisions. A large-scaled investigation is needed to obtain answers to unresolved issues and to strengthen the strategies for patients requiring long-term ECMO support.

## Figures and Tables

**Table 1:** Baseline characteristics of patients with acute respiratory distress syndrome on venovenous extracorporeal membrane oxygenation and the condition of patients before venovenous extracorporeal membrane oxygenation classified by the presence of COVID-19.

	Total (N = 45)	Non-COVID-19 (N = 24)	COVID-19 (N = 21)	*p* Value
Age	61 (54.00–67.00)	57 (53.25–65.75)	63 (54.50–67.50)	0.255
Sex, male	27 (60)	15 (62.5)	12 (57.1)	0.767
Body mass index (kg/m^2^)	22.7 (20.53–25.93)	23.71 (21.91–26.89)	21.74 (19.66–24.69)	0.144
Diabetes mellitus	22 (48.9)	10 (41.7)	12 (57.1)	0.376
Hypertension	20 (44.4)	10 (41.7)	10 (47.6)	0.769
Cardiovascular	7 (15.6)	5 (20.8)	2 (9.5)	0.422
COPD	7 (15.6)	5 (20.8)	2 (9.5)	0.422
Chronic kidney disease stage V	3 (6.7)	1 (4.2)	2 (9.5)	0.592
Malignancy or immunocompromised host	15 (33.3)	12 (50)	3 (14.3)	0.026
APACHE II score	18 (14–22.50)	19.5 (15.25–24.00)	15 (13–20)	0.121
SOFA score	9 (7.00–12.00)	9 (8.00–12.75)	8 (4.00–11.50)	0.033
Vasopressor-inotropic score	3.50 (0.00–21.25)	23.71 (21.91–26.89)	3.0 (0.00–10.00)	0.551
Charlson comorbidity index	3 (2.0–5.0)	3.5 (2.0–5.0)	3 (2.0–4.0)	0.256
Etiology of ECMO				-
COVID-19	21 (46.7)	0	21 (100)	
Bacterial/other pneumonia	11/4 (24.4/8.9)	11/4 (45.8/16.7)	-	
Aspiration pneumonitis	4 (8.9)	4 (16.7)	-	
Interstitial lung disease	3 (6.7)	3 (12.5)	-	
Other	3 (6.7)	3 (12.5)	-	
Mechanical ventilator setting before ECMO				
PEEP (cmH_2_O)	10 (7.75–10.00)	8.5 (6.00–10.00)	10 (8.00–12.00)	0.069
Volume control/pressure control	27/14	14/8	13/6	1.00
V_T_/PBW (mL/kg)	6.78 (6.15–7.83)	7.25 (6.15–8.03)	6.49 (6.17–7.61)	0.481
Blood gas analysis before ECMO				
PF ratio (mmHg)	59.50 (48.25–78.90)	58.9 (44.50–76.60)	61 (50.70–87.95)	0.488
pH	7.37 (7.21–7.42)	7.32 (7.17–7.38)	7.40 (7.31–7.45)	0.034
PO_2_ (mmHg)	60.70 (46.9–77.0)	57.1 (43.2–73.7)	62.0 (53.0–87.3)	0.120
PCO_2_ (mmHg)	51 (42–66)	63.2 (43.4–71.3)	44.0 (37.3–52.6)	0.006
HCO^3−^ (mmol/L)	25.9 (21.8–32.7)	25.9 (20.93–34.68)	25.5 (21.8–31.3)	0.903
SaO_2_ (%)	88.55 (79.47–94.28)	84.3 (64.9–93.1)	91.0 (85.3–96.4)	0.049

APACHE acute physiologic and chronic health evaluation; COPD chronic obstructive pulmonary disease; ECMO extracorporeal membrane oxygenation; IQR interquartile range; PEEP positive end expiratory pressure; SOFA sequential organ failure assessment.

**Table 2:** Disease course and prognosis of patients with acute respiratory distress syndrome on venovenous extracorporeal membrane oxygenation classified by the presence of COVID-19.

	Total (N = 45)	Non-COVID-19 (N = 24)	COVID-19 (N = 21)	*p* Value
Days from Diagnosis of COVID-19 to ECMO	11.00 (5.50–19.50)	-	11.00 (6.00–20.00)	-
Days from ICU admission to MV	0.00 (0.00–1.00)	0.00 (0.00–1.75)	0.00 (0.00–0.50)	0.132
Days from ICU admission to ECMO	3.00 (1.00–9.50)	2 (1.00–8.75)	4.0 (1.0–10.5)	0.492
Days from MV to ECMO	2.00 (0.00–7.00)	1.50 (0.00–6.00)	3.0 (1.00–10.50)	0.196
Mechanical ventilation day	30.00 (12.00–57.00)	18.50 (7.00–50.75)	38 (16.50–58.00)	0.219
ECMO day	15 (8.00–45.00)	10.5 (3.25–29.25)	28.0 (10.50–70.50)	0.018
Hospital day	47.00 (27.00–92.50)	41 (18.25–71.75)	59.0 (33.0–121.5)	0.082
Mechanical ventilator weaning off at discharge	18/22 (81.8)	8/9 (88.9)	10/13 (76.9)	1.0
ECMO weaning	24 (52.1)	12 (50.0)	12 (57.1)	0.767
28-day mortality	11 (24.4)	9 (37.5)	2 (9.5)	0.040
Hospital mortality	23 (51.1)	15 (62.5)	8 (38.1)	0.139
CRRT	20 (44.4)	13 (54.2)	7 (33.3)	0.231
CNS bleeding	1 (2.2)	1 (4.2)	0 (0)	1.00
UGI bleeding	9 (20.0)	3 (12.5)	6 (28.6)	0.267
pneumothorax	9 (20.0)	6 (25)	3 (14.3)	0.469
ECMO cannula related infection	2 (4.4)	1 (4.2)	1 (4.8)	1.000
Thrombosis of ECMO cannula	4 (8.9)	1 (4.2)	3 (14.3)	0.326

CNS central nervous system; CRRT continuous renal replacement therapy; ECMO extracorporeal membrane oxygenation; IQR interquartile range; UGI upper gastrointestinal.

**Table 3:** Univariate and multivariate Cox proportional hazard regression analysis for hospital mortality in patients with acute respiratory distress syndrome on venovenous extracorporeal membrane oxygenation.

	Unadjusted Hazrd Ratio	95% Confidence Interval	*p*-Value	Adjusted Hazard Ratio	95% Confidence Interval	*p*-Value
Age	1.046	0.995–1.099	0.075	1.068	1.010–1.130	0.022
Sex	0.885	0.363–2.101	0.782	1.374	0.472–4.004	0.560
CCI	1.429	1.138–1.794	0.002	1.405	1.041–1.896	0.026
SOFA	1.148	1.027–1.282	0.015	1.213	1.044–1.410	0.012
APACHE II	1.063	1.017–1.111	0.007			
PaO_2_/FiO_2_ ratio	0.998	0.985–1.011	0.742			
PEEP	0.880	0.782–0.992	0.036	1.014	0.879–1.170	0.847
V_T_/PBW	0.892	0.558–1.427	0.634			
COVID-19	0.325	0.124–0.849	0.022	0.350	0.110–1.115	0.076

APACHE acute physiologic and chronic health assessment; CCI Charlson comorbidity index; PBW predicted body weight; PEEP positive end expiratory pressure; SOFA sequential organ failure assessment.

## Data Availability

The datasets used and/or analyzed during the current study are available from the corresponding author on reasonable request.

## References

[1] Taubenberger J.K., Morens D.M. (2006). 1918 Influenza: The mother of all pandemics. Emerg. Infect. Dis..

[2] Golicnik A., Zivanovic I., Gorjup V., Berden J. (2023). Same but Different-ECMO in COVID-19 and ARDS of Other Etiologies. Comparison of Survival Outcomes and Management in Different ARDS Groups. J. Intensive Care Med..

[3] Cho H.J., Heinsar S., Jeong I.S., Shekar K., Li Bassi G., Jung J.S., Suen J.Y., Fraser J.F. (2020). ECMO use in COVID-19: Lessons from past respiratory virus outbreaks—A narrative review. Crit. Care.

[4] Ramanathan K., Antognini D., Combes A., Paden M., Zakhary B., Ogino M., MacLaren G., Brodie D., Shekar K. (2020). Planning and provision of ECMO services for severe ARDS during the COVID-19 pandemic and other outbreaks of emerging infectious diseases. Lancet Respir. Med..

[5] Davies A., Jones D., Bailey M., Beca J., Bellomo R., Blackwell N., Forrest P., Gattas D., Granger E., Herkes R. (2009). Extracorporeal Membrane Oxygenation for 2009 Influenza A(H1N1) Acute Respiratory Distress Syndrome. JAMA.

[6] Urner M., Barnett A.G., Bassi G.L., Brodie D., Dalton H.J., Ferguson N.D., Heinsar S., Hodgson C.L., Peek G., Shekar K. (2022). Venovenous extracorporeal membrane oxygenation in patients with acute COVID-19 associated respiratory failure: Comparative effectiveness study. BMJ.

[7] Ramanathan K., Shekar K., Ling R.R., Barbaro R.P., Wong S.N., Tan C.S., Rochwerg B., Fernando S.M., Takeda S., MacLaren G. (2021). Extracorporeal membrane oxygenation for COVID-19: A systematic review and meta-analysis. Crit. Care.

[8] Karagiannidis C., Slutsky A.S., Bein T., Windisch W., Weber-Carstens S., Brodie D. (2021). Complete countrywide mortality in COVID patients receiving ECMO in Germany throughout the first three waves of the pandemic. Crit. Care.

[9] Wendisch D., Dietrich O., Mari T., von Stillfried S., Ibarra I.L., Mittermaier M., Mache C., Chua R.L., Knoll R., Timm S. (2021). SARS-CoV-2 infection triggers profibrotic macrophage responses and lung fibrosis. Cell.

[10] George P.M., Wells A.U., Jenkins R.G. (2020). Pulmonary fibrosis and COVID-19: The potential role for antifibrotic therapy. Lancet Respir. Med..

[11] Russ M., Menk M., Graw J.A., Skrypnikov V., Hunsicker O., Rudat K., Weber-Carstens S., Francis R.C.E., Pickerodt P.A. (2022). COVID-19 Patients Require Prolonged Extracorporeal Membrane Oxygenation Support for Survival Compared with Non-COVID-19 Patients. Crit. Care Explor..

[12] Yaqoob H., Greenberg D., Huang L., Henson T., Pitaktong A., Peneyra D., Spencer P.J., Malekan R., Goldberg J.B., Kai M. (2023). Extracorporeal membrane oxygenation in COVID-19 compared to other etiologies of acute respiratory failure: A single-center experience. Heart Lung.

[13] The ARDS Definition Task Force (2012). Acute Respiratory Distress Syndrome: The Berlin Definition. JAMA.

[14] Ling R.R., Ramanathan K., Sim J.J.L., Wong S.N., Chen Y., Amin F., Fernando S.M., Rochwerg B., Fan E., Barbaro R.P. (2022). Evolving outcomes of extracorporeal membrane oxygenation during the first 2 years of the COVID-19 pandemic: A systematic review and meta-analysis. Crit. Care.

[15] Barbaro R.P., MacLaren G., Boonstra P.S., Combes A., Agerstrand C., Annich G., Diaz R., Fan E., Hryniewicz K., Lorusso R. (2021). Extracorporeal membrane oxygenation for COVID-19: Evolving outcomes from the international Extracorporeal Life Support Organization Registry. Lancet.

[16] Riera J., Alcántara S., Bonilla C., Fortuna P., Ortiz A.B., Vaz A., Albacete C., Millán P., Ricart P., Boado M.V. (2022). Risk factors for mortality in patients with COVID-19 needing extracorporeal respiratory support. Eur. Respir. J..

[17] Friedrichson B., Kloka J.A., Neef V., Mutlak H., Old O., Zacharowski K., Piekarski F. (2022). Extracorporeal membrane oxygenation in coronavirus disease 2019: A nationwide cohort analysis of 4279 runs from Germany. Eur. J. Anaesthesiol..

[18] Massart N., Guervilly C., Mansour A., Porto A., Flécher E., Esvan M., Fougerou C., Fillâtre P., Duburcq T., Lebreton G. (2023). Impact of Prone Position in COVID-19 Patients on Extracorporeal Membrane Oxygenation. Crit. Care Med..

[19] Xu Y., Chou Y.T., Wei X.G., Zhang S.F., Jiang F., Liu Y. (2022). ECMO combined with prone positioning strategies in COVID-19 respiratory distress syndrome. Perfusion.

[20] Papazian L., Schmidt M., Hajage D., Combes A., Petit M., Lebreton G., Rilinger J., Giani M., Le Breton C., Duburcq T. (2022). Effect of prone positioning on survival in adult patients receiving venovenous extracorporeal membrane oxygenation for acute respiratory distress syndrome: A systematic review and meta-analysis. Intensive Care Med..

[21] Peek G.J., Mugford M., Tiruvoipati R., Wilson A., Allen E., Thalanany M.M., Hibbert C.L., Truesdale A., Clemens F., Cooper N. (2009). Efficacy and economic assessment of conventional ventilatory support versus extracorporeal membrane oxygenation for severe adult respiratory failure (CESAR): A multicentre randomised controlled trial. Lancet.

[22] Fanelli V., Giani M., Grasselli G., Mojoli F., Martucci G., Grazioli L., Alessandri F., Mongodi S., Sales G., Montrucchio G. (2022). Extracorporeal membrane oxygenation for COVID-19 and influenza H1N1 associated acute respiratory distress syndrome: A multicenter retrospective cohort study. Crit. Care.

[23] Dreier E., Malfertheiner M.V., Dienemann T., Fisser C., Foltan M., Geismann F., Graf B., Lunz D., Maier L.S., Müller T. (2021). ECMO in COVID-19-prolonged therapy needed? A retrospective analysis of outcome and prognostic factors. Perfusion.

[24] Dave S.B., Rabinowitz R., Shah A., Tabatabai A., Galvagno S.M., Mazzeffi M.A., Rector R., Kaczorowski D.J., Scalea T.M., Menaker J. (2022). COVID-19 outcomes of venovenous extracorporeal membrane oxygenation for acute respiratory failure vs historical cohort of non-COVID-19 viral infections. Perfusion.

[25] Chen N., Zhou M., Dong X., Qu J., Gong F., Han Y., Qiu Y., Wang J., Liu Y., Wei Y. (2020). Epidemiological and clinical characteristics of 99 cases of 2019 novel coronavirus pneumonia in Wuhan, China: A descriptive study. Lancet.

[26] Wang D., Hu B., Hu C., Zhu F., Liu X., Zhang J., Wang B., Xiang H., Cheng Z., Xiong Y. (2020). Clinical Characteristics of 138 Hospitalized Patients with 2019 Novel Coronavirus–Infected Pneumonia in Wuhan, China. JAMA.

[27] Li Y., Wu J., Wang S., Li X., Zhou J., Huang B., Luo D., Cao Q., Chen Y., Chen S. (2021). Progression to fibrosing diffuse alveolar damage in a series of 30 minimally invasive autopsies with COVID-19 pneumonia in Wuhan, China. Histopathology.

[28] Gill J.R., Sheng Z.M., Ely S.F., Guinee D.G., Beasley M.B., Suh J., Deshpande C., Mollura D.J., Morens D.M., Bray M. (2010). Pulmonary pathologic findings of fatal 2009 pandemic influenza A/H1N1 viral infections. Arch. Pathol. Lab. Med..

